# Insane Kings

**Published:** 1897-05

**Authors:** 


### INSANE KINGS.

Bismarck, who opposed the marriage of Frederick (Crown Prince of Prussia), with the daughter of Queen Victoria (on the ground that she was a scion of the "crazy and scrofulous Guelphs") showed a prophetic insight into the results of those hereditary defects which Dr. E. S. Talbot, of Chicago, demonstrated to exist in aristocracies and royalties generally. The eccentricities of William II., of Germany, seem to fully justify Bismarck's objection. His recent performances are curiously like those of King Ludwig of Bavaria antecedent to that suicide and homicide which resulted in a great loss to medical science through the death of the cerebral anatomist Gudden. The socialist newspapers outline the artistic, architectural, sartorial, musical and pugilistic performances of William II. in a style that strikingly resembles the Suetonian sketch in 1884 of Ludwig's performances, and which testimony taken in 1886 by the Bavarian Lunacy Commission (Drs. Gudden, Hagen, Grashey and Hubrich) demonstrated to be correct. The stigmata of degeneracy notoriously present in William II. of Germany, the insanity of his grand-uncle Fredrick William IV. and that present in his paternal grandmother's ancestry lend emphasis to the fear Bismarck expressed of the taint in the Guelph-Wettin blood, which extends even to the inheritance of hemophilia. The *British Medical Journal* (a decade ago, anent the political complications consequent on the larynx cancer of Frederick) remarked that the short reign of a wise ruler stricken with a fatal disease is better than the long rule of a half-imbecile ruler affected with chronic disorder. The *British Medical Journal* here recognizes fully the difficulties, even in constitutional monarchies, of securing regencies in case of even notoriously insane kings. The sea-kings were able to ravage Christendom through the mental defects consequent on epilepsy of the Carlovingian and Merovingian dynasties. The lucid period of the paroxysmally excited paranoiac is peculiarly dangerous in this respect, as witness the cases of Wencelaus (Emperor of Germany and King of Bohemia), Charles VI. of France, Richard II. and Henry VI., of England, Eric XIV., of Sweden, Christian VII. of Denmark, and Ivan the Terrible, of Russia. Much stress has been laid on the case of Charles II., of Spain, who concluded the dynasty which ruled Spain for nearly four centuries; but the European difficulties resulting therefrom would probably have occurred on the extinction of the dynasty irrespective of the mental state of its last member. The instances of most importance were those of George III., Christian VII., of Denmark, and Ludwig, of Bavaria. George III., though the scion of a dynasty created by a parliament representative of the people and destitute of hereditary right, attempted throughout his reign to be an absolute king.

His shrewd grandfather gave him credit in boyhood for just sufficient intelligence to read to his mother. In mental make-up he resembled closely his famous insane ancestor, Ernest the Pious, of Hanover. His mother had filled him with religious cant just sufficient to make him conceal his liaisons in brutal fashion. He had an admiration for art of the artificial, weak, classic style; for music of the street band style; for drama of practical joke, realistic type, and for church architecture of packing-box type. In these proclivities he displayed less intellectual power than his morally insane son, George IV., than Christian VII., of Denmark, or than Ludwig of Bavaria. In all these particulars he closely resembled his descendant, William II., of Germany. George III. was several times violently insane and died chronically insane.

The country was ruled as a regency for several years by the moral imbecile afterward, George IV., who himself had delusions about having won the battle of Waterloo. George III. was, like all paranoiacs, most dangerous when least demonstrably insane. During his periods of seeming lucidity he had all the cunning, inventive, stupid egotism of the paranoiac and, as Green ("History of the English People") remarks, the shame of the darkest hours of English history lie wholly at his door. Lord Chatham, whose counsels might have thwarted much of this shame, was stricken down with gouty insanity during the critical period of the American troubles. The Americans were taxed in defiance of Chatham's denunciation of the unconstitutionality of this and King George III. ruled the country through the ministry formed by Chatham. Harsh laws, repellant to the spirit of the English common law, made death the penalty for almost all offenses against property or person, in order to swell the coffers of the King with goods confiscated from the felon.

George III., when he became demonstrably insane, was placed under the care of Dr. Willis, who kept a private insane hospital conducted on excellent principles. When the paroxysmal excitement quieted down, the tories asserted the king had recovered, and in this were supported by Dr. Willis and his son, and opposed by Dr. Warren. George III. was declared sane by the English judges. Dr. Willis was given a pension of £7,500 annually for twenty-one years, and his son one of £3,250 annually, for life. Insane paroxysms recurred from time to time. until it became demonstrated that the case was a chronic one, and a regency was established. George III. reigned fifty-eight years.

The same arbitrary characteristics and artistic tendencies. but with greater demonstrable sexual perversion and greater intellectual powers were legally shown to be present in Ludwig, of Bavaria, and Christian VII., of Denmark.

Christian VII. was coarser but less hypocritical in sexual debauchery than George III. He was equally suspicious, and while he had greater intellectual power than George III, in art, science, music and architecture, he was as inventively stupid in political matters. Neither of the two compared for political foresight with Ludwig of Bavaria, the patron of Wagner. Wagner would probably not have risen to the height he did, but for Ludwig, whose musical and artistic criticism of the performances he insisted on enjoying alone, prepared Wagner for the onslaught he later encountered.

